# Supplementary figures and images for: Network analysis of patterns and relevance of enteric pathogen co-infections among infants in a diarrhea-endemic setting
Source: PLoS Comput Biol. 2023 Nov 22;19(11):e1011624. doi: 10.1371/journal.pcbi.1011624 (PMC10664872; doi:10.1371/journal.pcbi.1011624)

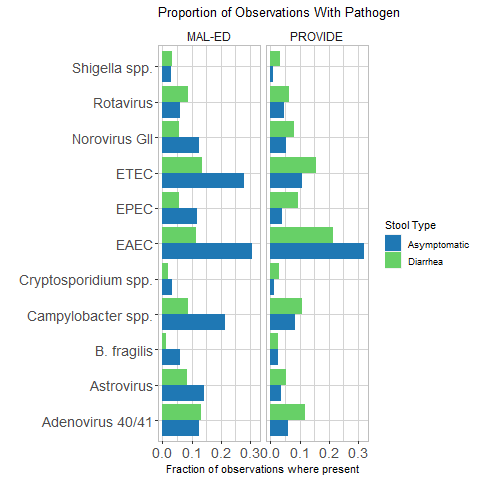

Supplement: S1 Fig — (PNG) [file pcbi.1011624.s001.png]

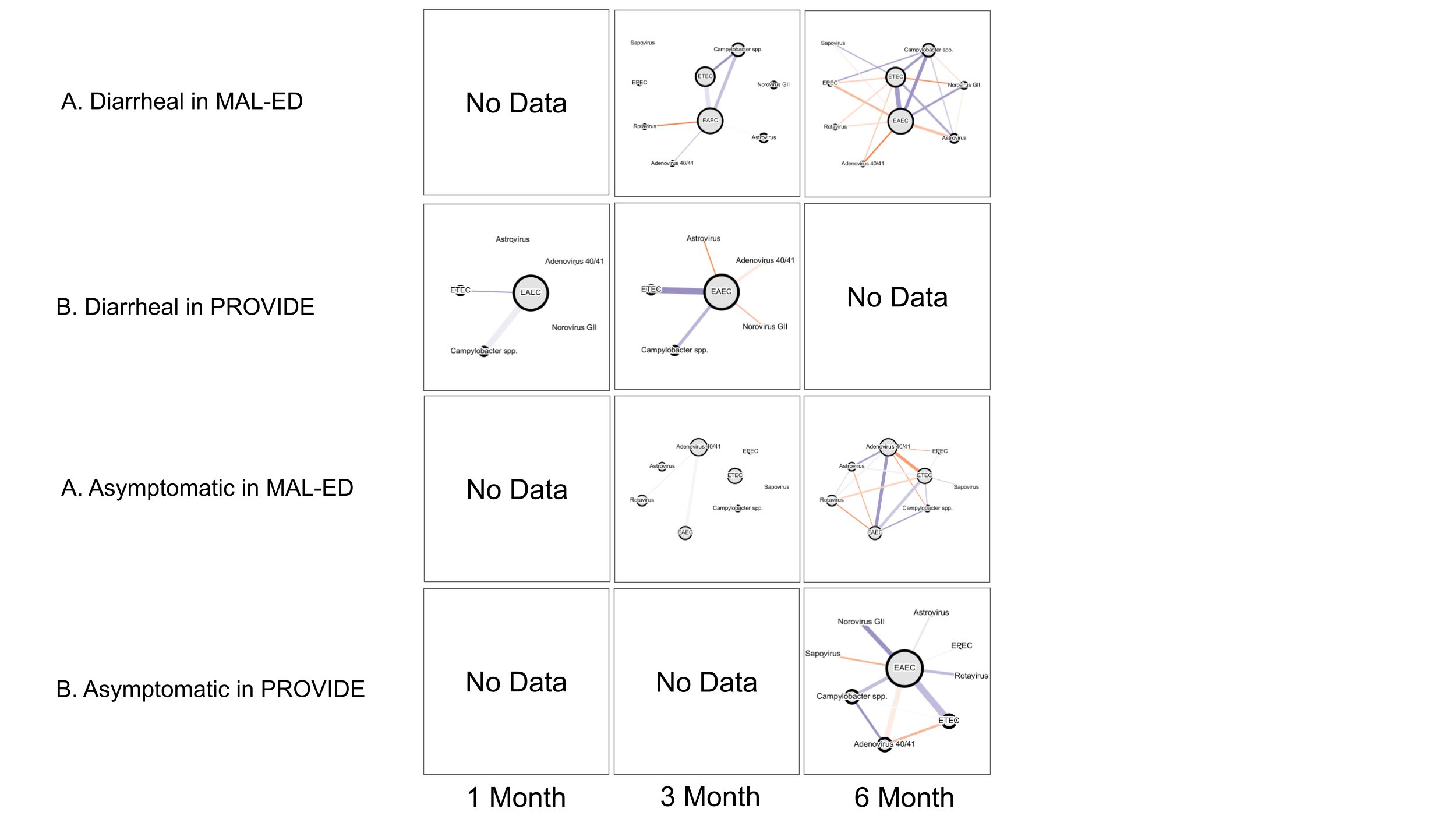

Supplement: S4 Fig — Each row represents a study and stool type, and each column represents a time window, either 1, 3 or 6 months. Networks were generated using the same methods as described above, using subsets of the original data based on the time of collection. Connections between pathogens are shown if they were observed to happen greater than ten times, and the saturation of color represents the distance from the null distribution average, where purple (orange) means the co-occurrences occurred higher (lower) than the null distribution, gray indicates the co-occurrences appeared at a rate similar to the average in the null distribution. (PNG) [file pcbi.1011624.s004.png]
