# Supplementary material for: Network analysis of patterns and relevance of enteric pathogen co-infections among infants in a diarrhea-endemic setting
Source: PLoS Comput Biol. 2023 Nov 22;19(11):e1011624. doi: 10.1371/journal.pcbi.1011624 (PMC10664872; doi:10.1371/journal.pcbi.1011624)
Supplement: S2 Fig — Includes top 10% of all pathogen pairs, ranked by the percentile’s distance from 0.5. With 435 possible combinations, 42 pairs make up the top 10% possible. (PDF) [file pcbi.1011624.s002.pdf]

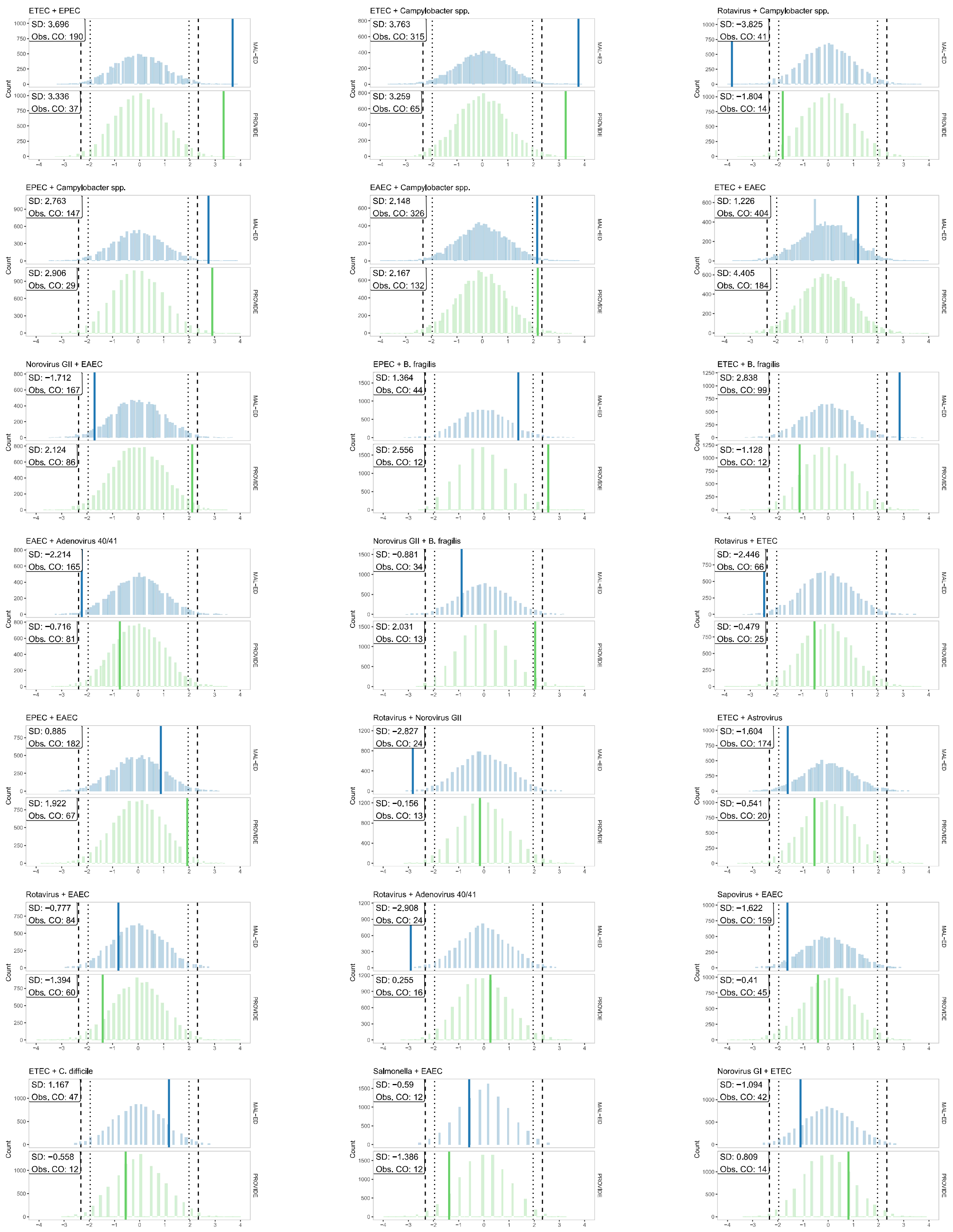

Figure S2: All null ensemble distributions from the configuration model of asymptomatic stools. An extension of Figure 2 to include the top 10% of all pathogen pairs, ranked by the percentile's distance from 0.5. With 435 possible combinations, 43 pairs make up the top 10% possible. Only 42 appeared > 10 times. Showing the top 21 out of 42 pairs.

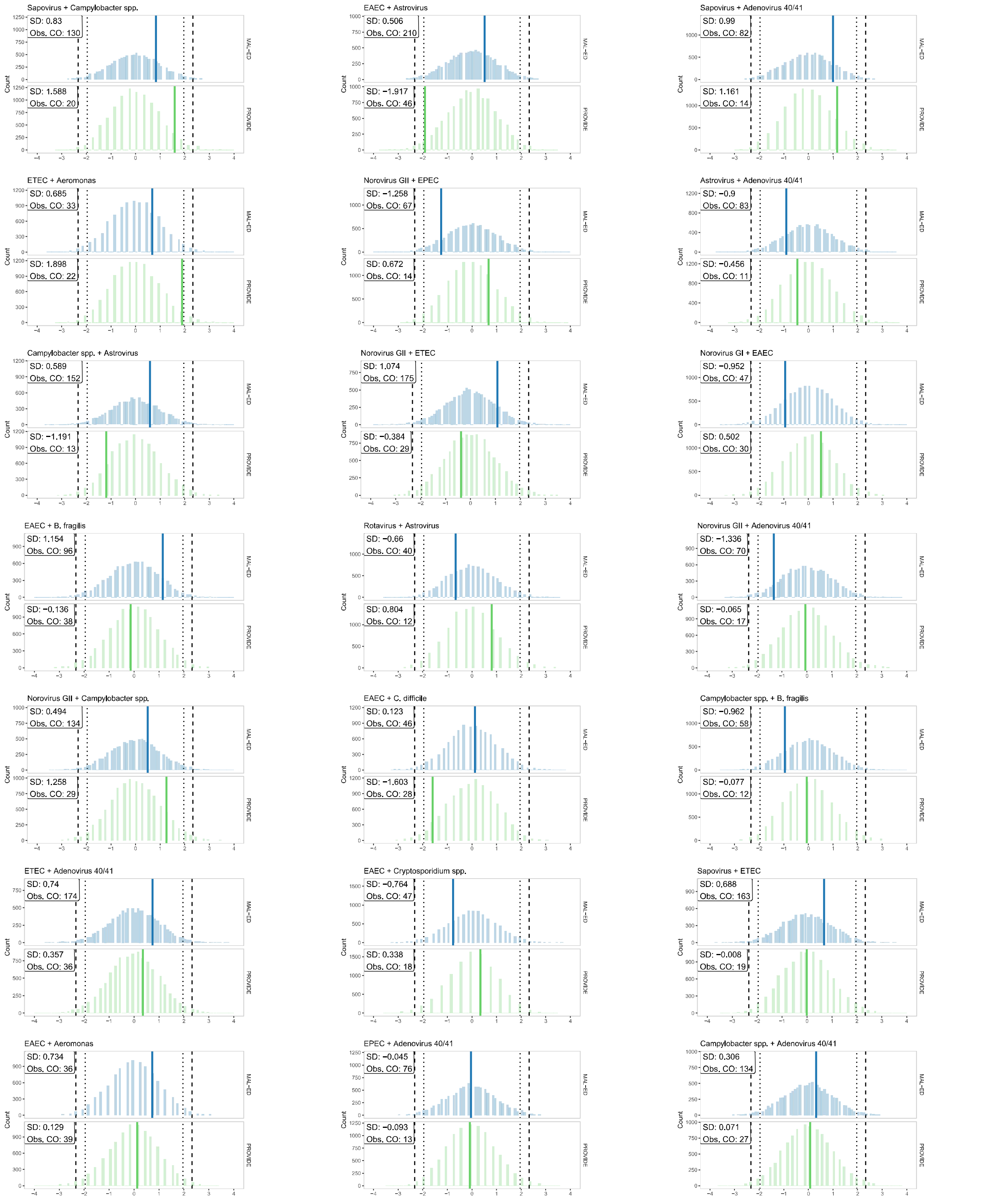

Figure S2 (cont.) : Continuation of the Figure S2 to show top 22 to 42 pairs.
