# Supplementary material for: Network analysis of patterns and relevance of enteric pathogen co-infections among infants in a diarrhea-endemic setting
Source: PLoS Comput Biol. 2023 Nov 22;19(11):e1011624. doi: 10.1371/journal.pcbi.1011624 (PMC10664872; doi:10.1371/journal.pcbi.1011624)
Supplement: S3 Fig — Includes top 10% of all pathogen pairs, ranked by the percentile’s distance from 0.5. With 520 possible combinations, 52 pairs make up the top 10% possible. (PDF) [file pcbi.1011624.s003.pdf]

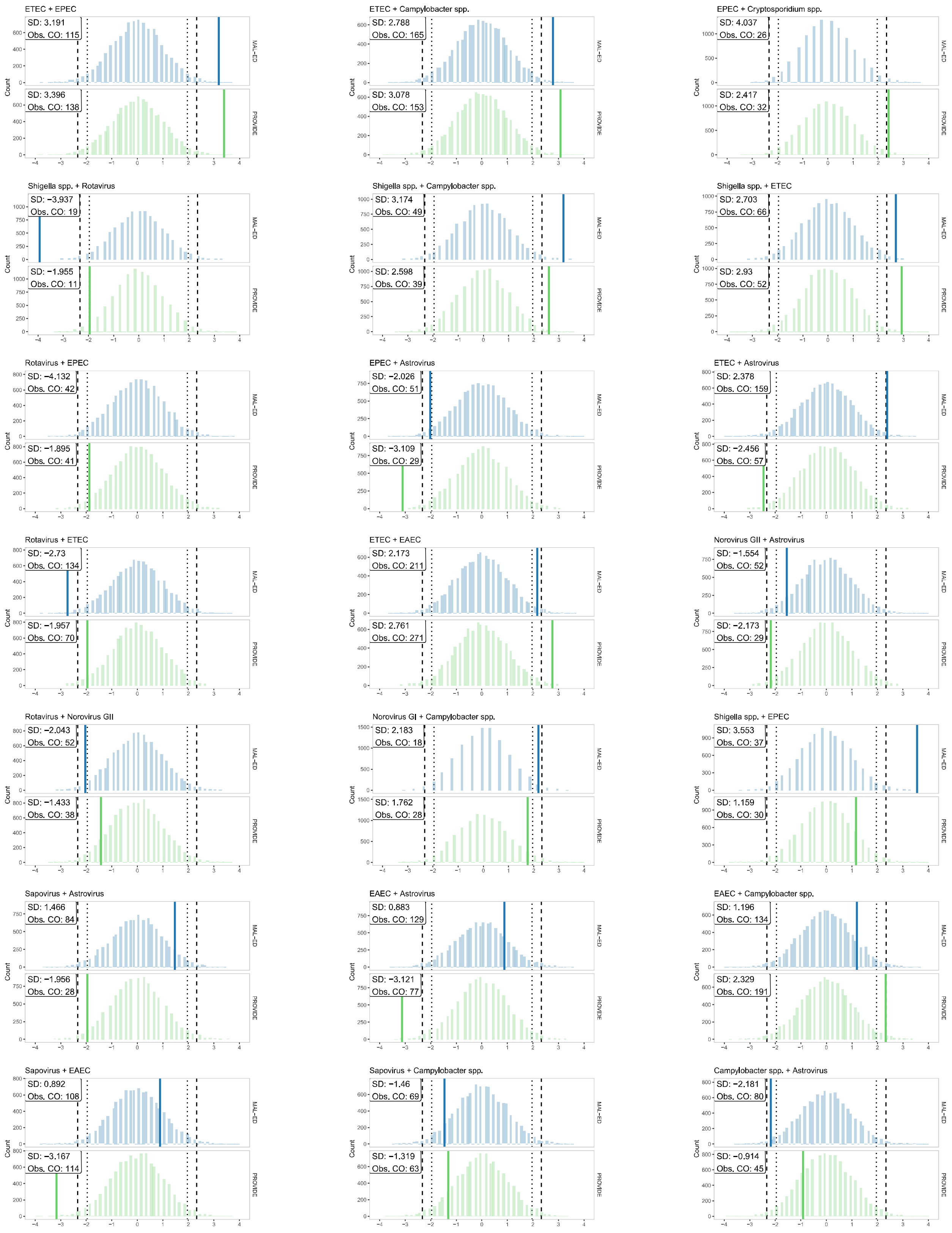

Figure S3: All null ensemble distributions from the configuration model of diarrheal stools. An extension of Figure 3 to include the top 10% of all pathogen pairs, ranked by the percentile's distance from 0.5. With 435 possible combinations, 43 pairs make up the top 10% possible. Showing the top 21 out of 43 pairs.

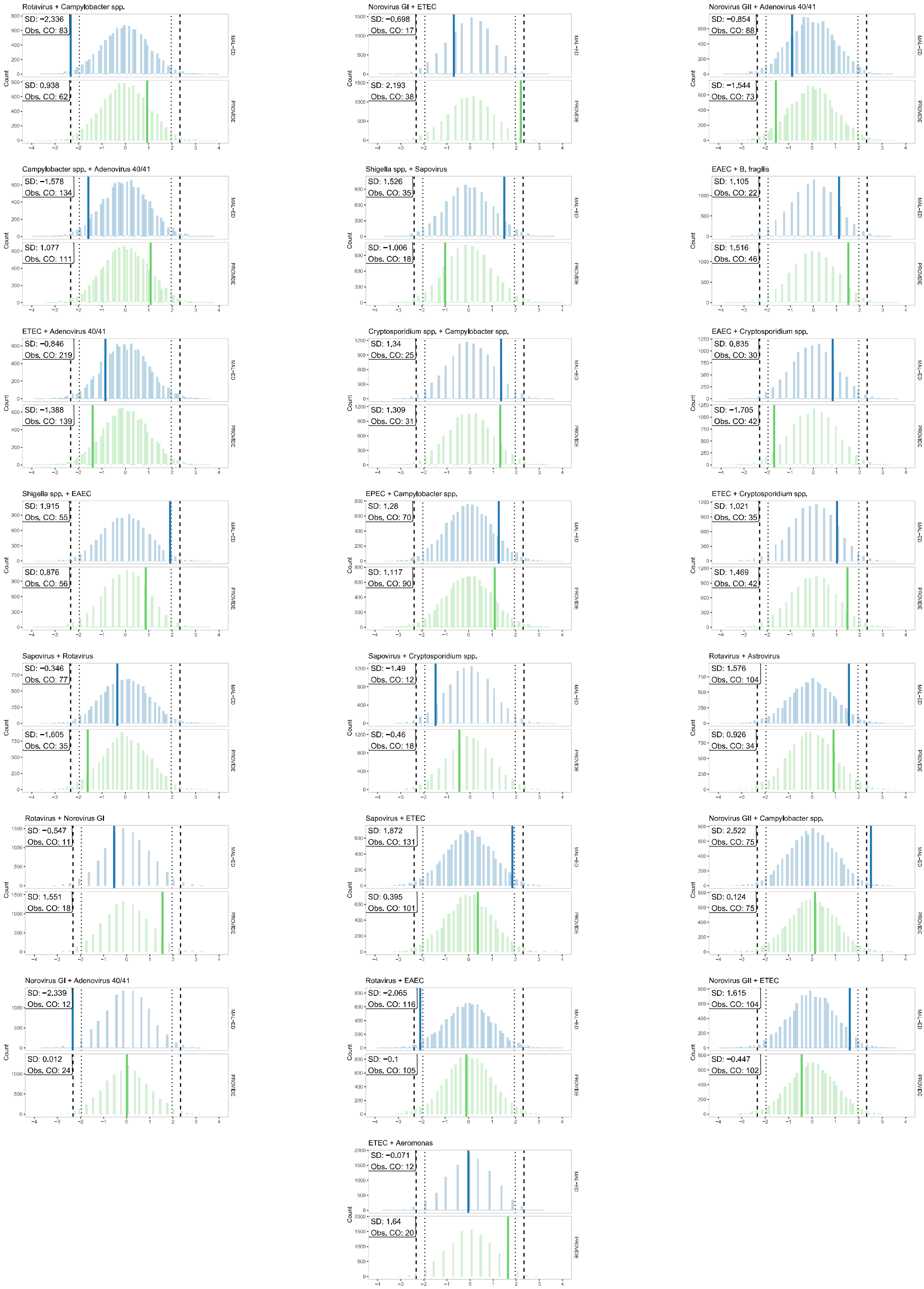

Figure S3(cont.): Continuation of the Figure S3 to show top 22 to 43 pairs.
