# Supplementary material for: Network analysis of patterns and relevance of enteric pathogen co-infections among infants in a diarrhea-endemic setting
Source: PLoS Comput Biol. 2023 Nov 22;19(11):e1011624. doi: 10.1371/journal.pcbi.1011624 (PMC10664872; doi:10.1371/journal.pcbi.1011624)
Supplement: S5 Fig — HOLMES is a generalization of χ2 test which compares pairs and higher-order groups for significant interactions in presence/absence data. Both HOLMES and our rewiring method control pathogen prevalence (marginal of a pathogen incidence over all samples) but only ours control for the inverse marginal of pathogens per stool (number of positives per stool over all pathogens). This difference means that while both methods generally agree on insignificant pairs and agree about four times more than expected at random on significant pairs, there is quite a bit of variability in other pairs found significant. We also note that, with its relaxed assumptions, HOLMES can then easily test for interactions beyond pairs and find only significant interactions in asymptomatic stools. (PDF) [file pcbi.1011624.s005.pdf]

A. Number of pathogen pairs found significant

B. Higher-order groups found significant

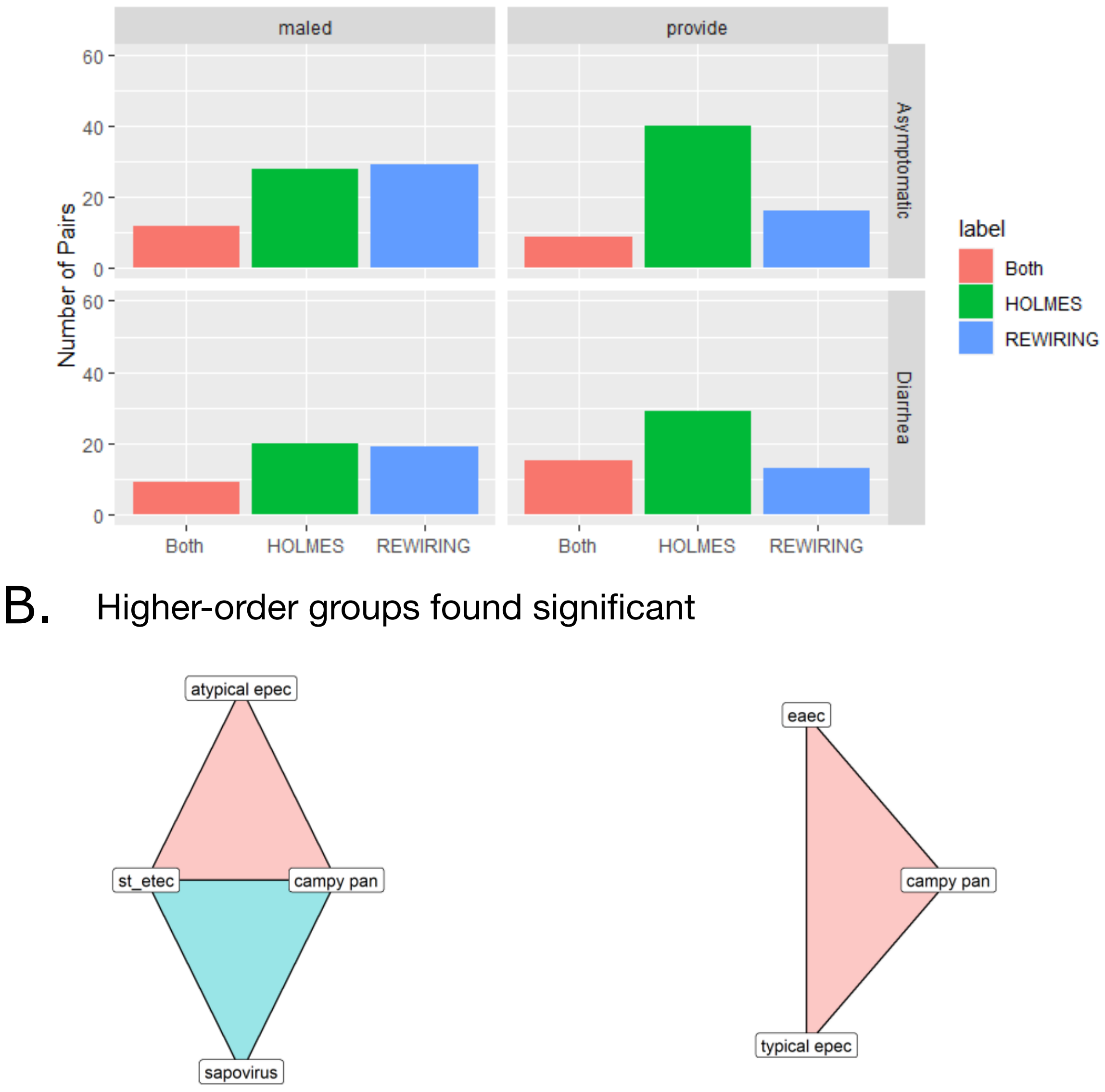

Asymptomatic in PROVIDE

Diarrheal in PROVIDE
