# Supplementary material for: Network analysis of patterns and relevance of enteric pathogen co-infections among infants in a diarrhea-endemic setting
Source: PLoS Comput Biol. 2023 Nov 22;19(11):e1011624. doi: 10.1371/journal.pcbi.1011624 (PMC10664872; doi:10.1371/journal.pcbi.1011624)
Supplement: S1 Table — (PDF) [file pcbi.1011624.s006.pdf]

Rank	Pathogen Pair	Number (%) of type-specific stools with pathogen pair	
		MAL-ED ($N = 2167$)	PROVIDE ($N = 1558$)
1	ETEC + EPEC	190 (8.7)	37 (2.4)
2	ETEC + *Campylobacter* spp.	315 (14.5)	65 (4.2)
3	Rotavirus + *Campylobacter* spp.	41 (1.9)	14 (0.9)
4	EPEC + *Campylobacter* spp.	147 (6.8)	29 (1.9)
5	EAEC + *Campylobacter* spp.	326 (15.0)	132 (8.5)
6	ETEC + EAEC	404 (18.6)	184 (11.8)
7	Norovirus GII + EAEC	167 (7.7)	86 (5.5)
8	EPEC + *B. fragilis*	44 (2.0)	12 (0.8)
9	ETEC + *B. fragilis*	99 (4.6)	12 (0.8)
10	EAEC + Adenovirus 4041	165 (7.6)	81 (5.2)
11	Norovirus GII + *B.fragilis*	34 (1.6)	13 (0.8)
12	Rotavirus + ETEC	66 (3.0)	25 (1.6)

Table S1: Top 12 pathogen pairs in asymptomatic stools, ranked by their deviation from the ensemble of random graphs, showing the number of co-occurrences and the occurrence as a percent out of asymptomatic stools.
