# Supplementary material for: Network analysis of patterns and relevance of enteric pathogen co-infections among infants in a diarrhea-endemic setting
Source: PLoS Comput Biol. 2023 Nov 22;19(11):e1011624. doi: 10.1371/journal.pcbi.1011624 (PMC10664872; doi:10.1371/journal.pcbi.1011624)
Supplement: S2 Table — (PDF) [file pcbi.1011624.s007.pdf]

Rank	Pathogen Pair	Number (%) of type-specific stools with pathogen pair	
		MAL-ED ($N = 717$)	PROVIDE ($N = 763$)
1	ETEC + EPEC	115 (16.0)	138 (18.1)
2	ETEC + *Campylobacter* spp.	165 (23.0)	153 (20.1)
3	EPEC + *Cryptosporidium* spp.	26 (3.6)	32 (4.2)
4	*Shigella* spp. + Rotavirus	19 (2.6)	11 (1.4)
5	*Shigella* spp. + *Campylobacter* spp.	49 (6.8)	39 (5.1)
6	*Shigella* spp. + ETEC	66 (9.2)	52 (6.8)
7	Rotavirus + EPEC	42 (5.9)	41 (5.4)
8	EPEC + Astrovirus	51 (7.1)	29 (3.8)
9	ETEC + Astrovirus	159 (22.2)	57 (7.5)
10	Rotavirus + ETEC	134 (18.7)	70 (9.2)
11	ETEC + EAEC	211 (29.4)	271 (35.5)
12	Norovirus GII + Astrovirus	52 (7.3)	29 (3.8)

Table S2: Top 12 pathogen pairs in asymptomatic stools, ranked by their deviation from the ensemble of random graphs, showing the number of co-occurrences and the occurrence as a percent out of diarrheal stools.
