# Supplementary material for: Network analysis of patterns and relevance of enteric pathogen co-infections among infants in a diarrhea-endemic setting
Source: PLoS Comput Biol. 2023 Nov 22;19(11):e1011624. doi: 10.1371/journal.pcbi.1011624 (PMC10664872; doi:10.1371/journal.pcbi.1011624)
Supplement: Attachment — Submitted filename: Colgate_AnswersToReviewers.pdf [file pcbi.1011624.s008.pdf]

Manuscript #PCOMPBIOL-D-23-00087—Network analysis of patterns and relevance of enteric pathogen co-infections among infants in a diarrhea-endemic setting

August 1, 2023

Dear Editor and Reviewers,

We thank you for your time and effort. We are particularly grateful that the goal of the paper was well understood and that the manuscript was judged to be interesting and innovative.

Altogether, your comments helped clarify many aspects of our manuscript. They also encouraged us to better frame the objective of the methods and run supplementary analyses. We have made clarifying revisions throughout and added several new analyses as supplementary figures. The goal of this structure is to focus the main text on our general approach to identifying significant co-occurrences and use supplementary figures to investigate the role of age and of higher-order co-occurrences. We particularly want to draw your attention to our new supplementary analyses:

- Age-controlled analyses,
- Comparison with another, less constrained but higher-order, network methods.

We provide a point-by-point response below, and a `diff` version of the manuscript to highlight the modifications.

The authors.

Reviewer A

Comment A.1

Interesting to read this work. Please add and compare with some state-of-the art methods with your proposed pipelines.

Reply A.1

We thank the Reviewer for their positive assessment of our work.

Action taken A.1

In our supplementary files, we have added a comparison with another analysis tool for networked co-occurrence. This new tool does not control for data distributions as much as we do, and therefore differs from ours in its details. Based on that difference, we do expect the methods to disagree around very high and low prevalence pathogens, yet the methods still agree on several pairs of pathogens. Obviously, there are no benchmarks for these methods, and we believe that having multiple tools operating on different assumptions can help better identify pathogen pairs with surprising co-occurrence statistics.

Reviewer B

Comment B.1

This manuscript reports on an analysis of enteric coinfections among infants to identify if specific coinfections occurred more often than would be expected by random chance. This is an important addition to the literature since diarrheal pathogens are often assessed individually despite the ubiquity of coinfections, for which their relevance is unknown. The use of network modeling to maintain pathogen prevalence and pathogen richness is innovative and appropriate. The manuscript is well written.

Reply B.1

We thank the Reviewer for their positive assessment of our work.

Comment B.2

The primary limitation is that the analysis does not account for the age structure of individual pathogen prevalences. Therefore, it doesn't account for the fact that coinfections would be expected to be more common for pathogens that have similar age distributions (even if infections with the pathogens are completely independent). For example, Shigella and rotavirus occur together less frequently than expected, but this is could be due to the fact that rotavirus is on average in younger children while Shigella is in older children. Differences in results between the two studies may also be affected by the age distribution of available and analyzed samples since non-diarrheal stools were collected in the first 6 months in PROVIDE when infections were less common.

Reply B.2

We agree completely. There is a key caveat here that indirect interactions due to age of the child are still of interest.

For example, it is interesting that our method picks up the negative correlations between Shigella and rotavirus used as an example. These indirect interactions can happen through age, nutrition, location, environment, etc., some of which are known and others unknown. The former, like Shigella and rotavirus, can be used as benchmarks for our methods; while the latter might lead to interesting future work that controls for different factors such as age, nutrition, location, etc.

Action taken B.2

We better explained the pipeline we envision and alternate forms of analysis: Starting from a network containing all data as we do in the main text, followed by networks constrained for different covariates of interest like age in supplementary figure S9. In these figures, stool samples are broken into 1 month, 3 month, and 6 month subsets according to the age of the child at stool collection. By controlling for age, we can see which pathogens might be found together at higher or lower rates in younger children. This is a useful output if we see pathogens that co-infect at rates higher than expected, especially if we expect to observe them at different ages of the child. In addition to controlling for age, we also explained in the discussion that the same methodology can be used for other environmental factors, such as location and socioeconomic status.

Comment B.3

Second, the analysis of the association between coinfections and diarrhea is limited since it is an ecological analysis (i.e. certain coinfections may be associated with more days of diarrhea overall, but it's not necessarily the case that the coinfections caused the diarrhea or even that the diarrhea occurred during the coinfections. These associations are likely confounded by host/environmental factors). Since data on pathogens during specific diarrhea episodes is known, this analysis could be done at the level of the diarrhea episode (i.e. are certain co-infections during diarrhea associated with longer duration of that episode). This analysis would also need to be adjusted for confounding factors like age, SES, malnutrition, and other factors associated with children having infections and more severe diarrhea. This might be too much for this manuscript. The novelty of this work is in the network analysis; I would recommend focusing on the primary results that limit the analysis to understanding whether coinfections occur more likely than would be expected by chance, incorporating the age structure of individual pathogen prevalence. Conclusions about which pathogens caused diarrhea (since pathogen quantities are not incorporated) or whether coinfections cause more diarrhea in general are overinterpretations of these analyses.

Reply B.3

We agree about the limits of ecological analysis simply correlating diarrheal outcomes with absence/presence data focused on certain co-infections. We also agree with the risk of attempting too much in a single paper. That being said, we really wish to help motivate as much future work as possible and hope we can find a compromise.

Action taken B.3

We have limited the results to the pathogen pairs identified using the configuration method. The relationship with diarrheal episodes is moved to the discussion. This more speculative analysis aims to outline the real-world implications, but needs to be contextualized with the caveats mentioned by the reviewer. The figure showing the relationship between co-infections and episode duration is now supplementary figure S4.

Comment B.4

Author summary (and throughout): suggest toning down causal language: "co-infection increases the number of days with diarrhea"

Action taken B.4

We have revised the text to be more observational than causal.

Comment B.5

M. tuberculosis is not an enteric pathogen (though it can be detected in stool because children cough and swallow). It would make the most sense to remove that from the analysis. B. fragilis is also not a pathogen (not associated with diarrhea) and I would suggest removing the use of "Enterotoxigenic Bacteroides fragilis."

Reply B.5

Thank you for your comment. Toxin-carrying *B. fragilis* was included in the TAC panel and in our analyses due to its established association with childhood diarrhea, please see references below:

- Wick EC, Sears CL. Bacteroides spp. and diarrhea. Curr Opin Infect Dis. 2010 Oct;23(5):470-4. doi: 10.1097/QCO.0b013e32833da1eb. PMID: 20697287; PMCID: PMC3079340.
- Sears CL, Islam S, Saha A, Arjumand M, Alam NH, Faruque AS, Salam MA, Shin J, Hecht D, Weintraub A, Sack RB, Qadri F. Association of enterotoxigenic Bacteroides fragilis infection with inflammatory diarrhea. Clin Infect Dis. 2008 Sep 15;47(6):797-803. doi: 10.1086/591130. PMID: 18680416; PMCID: PMC3045827
- Pathela P, Hasan KZ, Roy E, Alam K, Huq F, Siddique AK, Sack RB. Enterotoxigenic Bacteroides fragilis-associated diarrhea in children 0-2 years of age in rural Bangladesh. J Infect Dis. 2005 Apr 15;191(8):1245-52. doi: 10.1086/428947. Epub 2005 Mar 14. PMID: 15776370
- San Joaquin VH, Griffis JC, Lee C, Sears CL. Association of Bacteroides fragilis with childhood diarrhea. Scand J Infect Dis. 1995;27(3):211-5. doi: 10.3109/00365549509019011. PMID: 8539543

For *M. tuberculosis*, the prevalence of extra-pulmonary tuberculosis (TB) in the population of interest is unknown; however Bangladesh is classified as a high-burden country for TB. While, as the reviewer pointed out, one possible mechanism for the appearance of TB in enteric samples is via auto-inoculation from active pulmonary disease, potentially the ingestion of *M. bovis* in untreated milk products is a more relevant source of intestinal TB in young Bangladeshi children. We maintained the *M. tuberculosis* pathogen data in the analysis to preserve as much of the original dataset as possible. Furthermore, we expected positive detections of *M. tuberculosis* to be primarily sub-clinical and we speculate that while the pathogen may not be causally related to disease outcomes, the presence of *M. tuberculosis* may contribute to increased risk of enteric (and other) infections. It's also possible there's an unrecognized burden of intestinal TB in this population given the lack of specificity in symptoms and challenges with differential diagnoses. Finally, one strength of our approach is our focus on pathogen pairs co-occurring on the extremes of the distribution: pathogen pairs co-occurring as expected at random are de-emphasized in the analysis, which includes pairs with *M. tuberculosis* in this case. Please see additional references to support maintaining *M. tuberculosis* in the analysis:

- Nazneen A, Tarannum S, Chowdhury KIA, Islam MT, Islam SMH, Ahmed S, Banu S, Islam MS. Implementation status of national tuberculosis infection control guidelines in Bangladeshi hospitals. PLoS One. 2021 Feb 16;16(2):e0246923. doi: 10.1371/journal.pone.0246923. PMID: 33592049; PMCID: PMC7886225.
- Choi EH, Coyle WJ. Gastrointestinal Tuberculosis. Microbiol Spectr. 2016 Dec;4(6). doi: 10.1128/microbiolspec.TNMI7-0014-2016. PMID: 28084201.
- Debi U, Ravisankar V, Prasad KK, Sinha SK, Sharma AK. Abdominal tuberculosis of the gastrointestinal tract: revisited. World J Gastroenterol. 2014 Oct 28;20(40):14831-40. doi: 10.3748/wjg.v20.i40.14831. PMID: 25356043; PMCID: PMC4209546.

Comment B.6

3. The rotavirus coinfection results that differ across studies may also be due to age – if vaccination increases the average age of rotavirus infection, it is more likely that vaccinated children will have coinfections with rotavirus and bacteria, since bacteria also more often occur at older ages. It is not clear how these results support the conclusion that rotavirus is likely the causative agent of infection especially in the absence of vaccination. The assumption that the absence of other pathogens means rotavirus is the likely etiology is unstated. This should be moved from the results to the discussion with further explanation. Importantly, the analysis does not incorporate pathogen quantity which is a strong predictor of whether a pathogen present is the etiology or not. This information should be incorporated if conclusions are made about rotavirus being the likely etiology or not (not just whether coinfections were present).

Reply B.6

We see the reviewer's point about the relationship with rotavirus to diarrhea outcomes. Therefore, we are moving Figure 6 to the supplementary figures and now discuss this further in the Discussion section. We have also included an additional supplementary figure (S9) showing the results when partitioning the data by age, as well as an explanation of some of the limitations in doing so in the Discussion, namely the size of the data set.

As to the reviewer's second point, we agree that quantification is an important addition to future analyses, as acknowledged in our limitations to the analysis in the Discussion section. We did not include the quantitative aspects of the PCR data in this initial application of the network model due to the uncertainty of assigning biological relevance to CT values for different pathogens. This is a limitation we would want to address in future work.

Action taken B.6

We have moved Figure 6 to supplemental figures and discuss it in a more speculative manner in the Discussion.

Comment B.7

Figure S3 reference is missing in the text.

Action taken B.7

Added.

Comment B.8

Figure 5: a simple bar chart might be easier to read here. The comparison in the text of proportion diarrheal for each pathogen pair should be made to the proportion diarrheal for the included individual pathogens, not diarrhea overall. Perhaps this could be incorporated into the figure?

Reply B.8

We thank the reviewer for your feedback on this. We have updated the figure. We now discuss in the text the relative risk of diarrhea for a co-infection. This is measured as the rate of co-infection in diarrhea compared to the rate of co-infection in asymptomatic stools.

Action taken B.8

Figure 5 has been updated to a simple bar chart. Instead of showing the fraction of stools that are diarrheal with the co-infection, the figure now compares the rate of confection in diarrheal stools to that of asymptomatic stools. The text now compares and discusses this risk metric.

Comment B.9
Alternatively, the analysis of the proportion of stools for each co infection that are diarrheal is confounded by the arbitrary ages at which non-diarrheal stool samples were collected (particularly, non-diarrheal stools in PROVIDE only in the first 6 months) and the age distribution of pathogen prevalence. Rather than the proportion diarrheal, a relative risk measure (proportion of diarrheal stools with coinfection / proportion of non-diarrheal stools with coinfection) would account for the different denominators/sampling schemes between diarrheal and non-diarrheal stools

Reply B.9
We agree with the Reviewer.

Action taken B.9
See action taken for comment B.8. The Figure now shows the percent of diarrheal stools with co-infection over the percent of asymptomatic stools with co-infection.

Comment B.10
The sampling strategy in PROVIDE (mentioned to have been done for a separate analysis) needs to be completely described in the methods. Why were only vaccinated children included from PROVIDE?

Reply B.10
Thank you for this suggestion. We have explained the sampling strategy in greater detail under "Study design and participants" in the Methods section of the paper. In short, the sub-set of PROVIDE children included in the analyses were those with complete pathogen data at all time points, which included only vaccinated children. The sub-set was originally chosen for a separate analysis looking at immune markers and breakthrough rotavirus diarrhea in vaccinated children.

Action taken B.10
Revised "Study design and participants" in Methods section to better explain the sampling strategy.

Comment B.11
The last paragraph of the results is an over interpretation of the data presented due to the concerns described above.

Action taken B.11
Removed.

Comment B.12
Figure 7 – pathogens are not defined the same as earlier in the paper (aEPEC and tEPEC separate, for example). Also, there are nodes for both ETEC and LT and ST ETEC, separately? It would be nice to see the results for aEPEC and tEPEC separately throughout the paper since the relevance of aEPEC as a pathogen is unclear.

Reply B.12
Thank you for your careful attention to Figure 7. Inadvertently, the version of the figure reviewed was based on a

previous version of the analysis not included in this paper. In the final analysis, we dropped aEPEC because, as you mention, its clinical relevance is uncertain. We retained tEPEC and relabeled it as EPEC, so in our final analysis, these are separated, as suggested. Also in our final analysis we combined ST and LT ETEC into "ETEC", because we were getting an amplified result for each separately due to cross-detection with identical genetic components in each strain, except the toxins.

Action taken B.12
This figure has been updated to show the correct version, only showing EPEC and ETEC.

Comment B.13
This may be beyond the scope of the paper, but it would be nice to know how generalizable these results are to other sites in MAL-ED

Action taken B.13
We now clarify in the Discussion that, as a methods paper, we focused on one location with more than one study to evaluate whether our approach is sensitive enough to pick up differences not based on geography. Future work will extend this analysis to other sites in MAL-ED.

Comment B.14
Table 1: H. pylori has a typo

Action taken B.14
Fixed.

Comment B.15
Fig 2 and 3 legends – there are typos around the bounds ¿ 4 SDs (looks like PDF did not render correctly)

Action taken B.15
Fixed.
