# Supplementary material for: Network analysis of patterns and relevance of enteric pathogen co-infections among infants in a diarrhea-endemic setting
Source: PLoS Comput Biol. 2023 Nov 22;19(11):e1011624. doi: 10.1371/journal.pcbi.1011624 (PMC10664872; doi:10.1371/journal.pcbi.1011624)
Supplement: Attachment — Submitted filename: Review.pdf [file pcbi.1011624.s009.pdf]

Review of PCOMPBIOL-D-23-00087R1for *PLOS Computational Biology*

The authors have done an excellent job of addressing previous critiques and the manuscript is stronger as a result of the edits. I do have a few minor comments:

- I think that it's important that the authors provide additional background information on the variety of reasons why children may carry multiple enteric pathogens without symptoms. I think this would improve the discussion section. See: Myron M. Levine , Roy M. Robins-Browne, Factors That Explain Excretion of Enteric Pathogens by Persons Without Diarrhea, *Clinical Infectious Diseases*, Volume 55, Issue suppl_4, December 2012, Pages S303–S311, https://doi.org/10.1093/cid/cis789
- Figure 4 seemed to be corrupted in the PDF and it's difficult to read
- Figure 5 also seemed to be corrupted in the PDF and was difficult to read
- The authors included Cryptosporidium in their Bacteria-Bacteria histogram of Figure 5, panel A. Cryptosporidium is a protozoan and it's not clear why it's included in this panel of the figure.
