# Supplementary material for: Network analysis of patterns and relevance of enteric pathogen co-infections among infants in a diarrhea-endemic setting
Source: PLoS Comput Biol. 2023 Nov 22;19(11):e1011624. doi: 10.1371/journal.pcbi.1011624 (PMC10664872; doi:10.1371/journal.pcbi.1011624)
Supplement: Attachment — Submitted filename: Colgate2023_Response.pdf [file pcbi.1011624.s010.pdf]

Manuscript #PCOMPBIOL-D-23-00087R1—Network analysis of patterns and relevance of enteric pathogen co-infections among infants in a diarrhea-endemic setting

October 7, 2023

Dear Editor and Reviewers,

We again thank you for your time and effort. We are grateful that the revisions were judged appropriate and have now made the final requested changes to the presentation.

We provide a point-by-point response below, and a `diff` version of the manuscript to highlight the modifications.

The authors.

Reviewer A

Comment A.1

Author's revised where its needed.

Reply A.1

We thank the Reviewer for their positive assessment of our revisions.

Reviewer B

Comment B.1

I like the addition of Figure 5 and the relative risks. Can you add confidence intervals to the relative risk estimates in the text? It also would be helpful if the RRs are compared to the RRs for the individual pathogens so that the reader can compare the RR for Shigella alone vs. Shigella + rotavirus, for example. I would also recommend adjusting the relative risk estimates for age, or at least acknowledging the potential for confounding by age in these estimates in the discussion.

Action taken B.1

We have added confidence intervals to the relative risks estimates in the main text. We mention how they were calculated as well as potential confounding factors to the relative risks.

Comment B.2

Many of the analyses (including figures) were simply moved to the discussion section and supplement, which is not appropriate (analyses should be in the results section). I maintain my recommendation to remove the diarrhea duration analysis because of the ecological nature (when you have data on individual episodes) and the likely confounding bias. I acknowledge that this was a lot of work, but it detracts from the novelty and impact of the rest of the paper due to the limitations detailed in my last review. Specifically, I would remove starting with "Regarding the association between top pathogen pairs and child health diarrheal outcomes at the individual level..." through "Given the high burden of diarrhea where this pair is present, interventions aimed at disrupting co-infection, as opposed to targeting mono-infection with either pathogen, may reduce the overall burden of enteric disease for infants." The potential impact on diarrhea outcomes/duration could be mentioned in the discussion section at a higher level without reporting these analyses.

Action taken B.2

We have removed the text in question and added a small note on this topic in the discussion.

Reviewer C

Comment C.1

I think that it's important that the authors provide additional background information on the variety of reasons why children may carry multiple enteric pathogens without symptoms. I think this would improve the discussion section. See: Myron M. Levine , Roy M. Robins-Browne, Factors That Explain Excretion of Enteric Pathogens by Persons Without Diarrhea, Clinical Infectious Diseases, Volume 55, Issue $suppl_4, December 2012, Pages S303–S311$.

Action taken C.1

We have added this reference when mentioning asymptomatic co-infections.

Comment C.2

Figure 4 and 5 seemed to be corrupted in the PDF and are difficult to read

Action taken C.2

We do not know what could have caused this but will work with the publishing team to make sure the figures do not get corrupted.

Comment C.3

The authors included Cryptosporidium in their Bacteria-Bacteria histogram of Figure 5, panel A. Cryptosporidium is a protozoan and it's not clear why it's included in this panel of the figure

Action taken C.3

The title of the panel was fixed to “Bacteria + others” and the caption clarifies that this concerns the entire dataset except viruses.
